# Critical Role of Energy Transfer Between Terbium Ions for Suppression of Back Energy Transfer in Nonanuclear Terbium Clusters

**DOI:** 10.1038/srep37008

**Published:** 2016-11-15

**Authors:** Shun Omagari, Takayuki Nakanishi, Yuichi Kitagawa, Tomohiro Seki, Koji Fushimi, Hajime Ito, Andries Meijerink, Yasuchika Hasegawa

**Affiliations:** 1 Graduate School of Chemical Sciences and Engineering, Hokkaido University, N13 W8, Kita-ku, Sapporo, Hokkaido 060–8628, Japan; 2 Faculty of Engineering, Hokkaido University, N13 W8, Kita-ku, Sapporo, Hokkaido 060–8628, Japan; 3 Debye Institute, Department of Chemistry, Utrecht University, Princetonplein 5, 3584 CC Utrecht, The Netherlands

## Abstract

Lanthanide (Ln(III)) complexes form an important class of highly efficient luminescent materials showing characteristic line emission after efficient light absorption by the surrounding ligands. The efficiency is however lowered by back energy transfer from Ln(III) ion to the ligands, especially at higher temperatures. Here we report a new strategy to reduce back energy transfer losses. Nonanuclear lanthanide clusters containing terbium and gadolinium ions, Tb_n_Gd_9−n_ clusters ([Tb_n_Gd_9−n_(μ-OH)_10_(butylsalicylate)_16_]^+^NO_3_^−^, *n* = 0, 1, 2, 5, 8, 9), were synthesized to investigate the effect of energy transfer between Tb(III) ions on back energy transfer. The photophysical properties of Tb_n_Gd_9−n_ clusters were studied by steady-state and time-resolved spectroscopic techniques and revealed a longer emission lifetime with increasing number of Tb(III) ions in Tb_n_Gd_9−n_ clusters. A kinetic analysis of temperature dependence of the emission lifetime show that the energy transfer between Tb(III) ions competes with back energy transfer. The experimental results are in agreement with a theoretical rate equation model that confirms the role of energy transfer between Tb(III) ions in reducing back energy transfer losses. The results provide a new strategy in molecular design for improving the luminescence efficiency in lanthanide complexes which is important for potential applications as luminescent materials.

Energy transfer (ET) plays a major role in various molecular photofunctional systems such as fluorescent biosensors^1^,^2^,^3^, photon upconverters^4^,^5^,^6^, and organic light-emitting diodes (OLEDs)^7^,^8^,^9^. The efficiency of ET is dependent on the distance between a donor and an acceptor with maximum effective range of 1 nm (in Dexter’s mechanism)^10^ or 10 nm (in Förster’s mechanism)^11^. Distance sensitivity is important in fluorescent biosensors since the efficiency of ET between a target biomolecule and a sensor changes according to the distance, allowing biological activities to be monitored through spectroscopic examination^12^. Closely assembled chlorophylls in a photosystem prompt fast ET and efficiently deliver the energy obtained through absorption of sunlight to a photosynthetic center^13^,^14^,^15^. In organic photovoltaics, ET between “energy relay dyes” and sensitizing dyes has been utilized to extend the absorption region for improvement of power conversion efficiency^16^,^17^. Photofunctional systems have been extensively studied in many scientific fields including chemistry, physics, and biology.

Effective use of energy transfer is also seen in luminescent lanthanide (Ln(III)) complexes, and the process is known as photosensitized energy transfer (PSET). The unique photophysical properties of Ln(III) ions include spectrally fixed, sharp, and long-lived luminescence arising from their characteristic 4f-4f transition^18^,^19^,^20^,^21^. However, efficient luminescent materials based on Ln(III) ions require photosensitization because of the small absorption coefficient of 4f-4f transitions. Photosensitization can be realized e.g. through energy transfer from organic ligands with a large ab-sorption coefficient (acting as an antenna), leading to intense lanthanide luminescence^18^,^22^,^23^. Much interest has therefore been shown in these Ln(III) complexes for their potential applications to luminescent materials such as those used in bioassays, spectral converters, and electroluminescent devices^24^,^25^,^26^,^27^,^28^,^29^,^30^,^31^. PSET efficiency is qualitatively known to be high when the energy level of an excited triplet state (T_1_) of a ligand is slightly higher than the (emitting) excited level of an Ln(III) ion. On the other hand, when T_1_ energy is too close, the reverse process of PSET, back energy transfer (BET), occurs^32^,^33^,^34^. Optimal excited state energies have been focused on in Ln(III) complexes to suppress BET and achieve high luminescence efficiency.

Green-luminescent Tb(III) complexes have been one of the most frequently reported examples of Ln(III) complexes with BET^29^,^33^,^34^,^35^,^36^,^37^. Experimental studies have revealed that the energy gap between the emitting level of Tb(III) ion (^5^D_4_ state) and T_1_ state of less than 1850 cm^−1^ promotes fast BET and low luminescence efficiency^34^,^37^,^38^. Consequently, Tb(III) complexes with high luminescence efficiency use organic ligands with high T_1_ state energy and therefore even higher singlet excited (S_1_) state energy, leading to absorption range shorter than 350 nm^34^,^39^,^40^. Expanding this range to a longer wavelength while maintaining high luminescence efficiency leads to a larger degree of freedom in molecular design, allowing incorporation of functionality, such as sensing, chirality, and self-assembly. A radically new strategy is required to suppress BET.

Herein, we propose a new method for suppressing BET in Tb(III) complexes: ET between Tb(III) ions (TbET). Photophysical processes are based on competitive kinetics^41^,^42^. Since both BET and TbET from a Tb(III) ion occur from the same ^5^D_4_ excited level, these two processes are competitive. When the contribution of TbET dominates over BET, BET is suppressed. The contribution of TbET can be increased by reducing the distance between Tb(III) ions to maximize the rate of TbET. An Ln(III) cluster would therefore be the best candidate because it consists of a core of oxygen-bridged Ln(III) ions, and hence the distance between Ln(III) ions is very short, usually about 3.7 Å^43^,^44^,^45^,^46^,^47^,^48^,^49^. We previously reported opto-magnetic properties of a nonanuclear Tb(III) cluster^50^,^51^. It was found that the structural features of this cluster, especially the closely assembled Tb(III) ions and the strong interaction between them, enhances the opto-magnetic property. The cluster is an ideal model to investigate the relationship between BET and TbET and to demonstrate the role of TbET in suppressing BET.

In this study, we introduced Gd(III) ions to nonanuclear Tb(III) clusters in order to investigate the relationship between TbET and luminescence efficiency (Fig. 1a). The energy level of an excited state of Gd(III) ions is high (>31500 cm^−1^), allowing Gd(III) ions to act as spacers between Tb(III) ions to modulate the TbET processes in otherwise identical clusters. A detailed theory on how TbET can lead to suppression of BET in a nonanuclear Tb(III) cluster model is given in the “Theoretical Background” section. Novel nonanuclear Ln(III) clusters containing Tb(III) and Gd(III) [Tb_n_Gd_9−n_(μ-OH)_10_(butylsalicylate)_16_]^+^NO_3_^−^ (*n* = 0, 1, 2, 5, 8, 9) (referred to as “Tb_n_Gd_9−n_ clusters”) were synthesized by complexation reaction of Tb(III)/Gd(III) nitrate salt and butylsalicylate. Their characteristic structures were evaluated using FAB-MS, XRD, and X-ray single crystal analysis. Emission spectroscopy, determination of quantum yield, and emission lifetime measurements were performed for the clusters in a chloroform solution. Temperature dependency of emission lifetimes was also determined for estimation of the activation energy of BET. The fundamental photophysics of Ln(III) clusters and their potential as new class of highly efficient luminescent materials are presented in this report.

## Results and Discussion

### Theoretical Background

**Figure 1:**
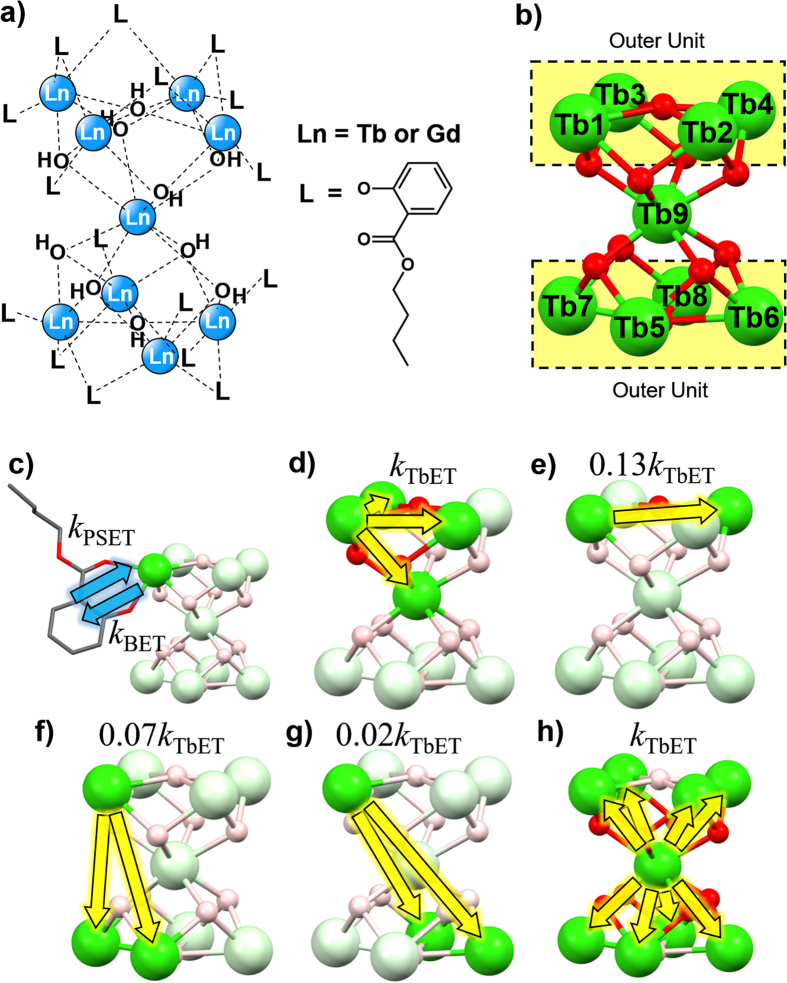
(**a**) Nonanuclear Tb(III) cluster (Tb_9_ cluster). (**b**) Labeling of Tb_9_ cluster. The center Tb(III) ion is denoted Tb9, and the Tb(III) ions in the outer unit are denoted Tb*m (m* = 1–8). (**c**) Energy transfer between Tb*m* and butylsalicylate ligand (only one ligand is depicted for clarity). (**d**) Energy transfer between Tb(III) ions from the point of view of Tb1 separated by 3.65 Å, (**e**) by 5.10 Å, (**f**) 5.64 Å, and (**g**) 7.10 Å. The same processes apply for all other Tb*m*. (**h**) Energy transfer between Tb(III) ions from the point of view of Tb9. The energy transfer rate constant is defined as *k*_TbET_ for two Tb(III) ions separated by 3.65 Å, and other Tb(III) ion pairs are defined relative to this rate constant according to Förster’s mechanism.

The theoretical background of the concept that back energy transfer (BET) can be suppressed using energy transfer between Tb(III) ions (TbET) in a nonanuclear Tb(III) cluster is provided in this section by using rate equations. The kinetic energy transfer processes in Tb_9_ clusters^50^ is depicted in Fig. 1b–h. The energy transfer processes in the cluster include photosensitized energy transfer (PSET), BET, and TbET. Although the closest Tb(III)-Tb(III) pair is less than 4 Å where multipolar and exchange interactions may contribute to the energy transfer rate^52^, calculation of this contribution does not significantly affect the results and conclusion of this section (see Supplementary Information S2 and Table S1). Therefore for the sake of discussion, the energy transfer rate constant between the closest pair of Tb(III) ions was defined as *k*_TbET_ and the transfer rates for the other pairs were calculated relative to *k*_TbET_ based on the simplest *R*^−6^ distance dependence of the dipole-dipole Förster mechanism. Tb(III) ions in the outer unit (Tb*m, m* = 1, 2, …, 8) are involved in PSET and BET with their coordinated butylsalicylate ligands. On the other hand, the center Tb(III) ion (Tb9) is coordinated only by oxygen atoms and is isolated from PSET and BET. The population density of Tb9 is solely dependent on the cycle of repetitive TbET to and from Tb(III) ions in the outer unit Tb*m* (energy migration). Based on these considerations, a system of differential equation in matrix form (Equation (1)) models the excited state dynamics of Tb_9_ clusters:


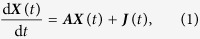


where ***X***(*t*) is the vector of population density of an excited singlet state (S1(*t*)), excited triplet state (T1(*t*)), excited Tb(III) ions in the outer unit (Tb*m*(*t*), *m* = 1, 2, …, 8), and the center Tb(III) ion (Tb9(*t*)) as shown in Equation (2)


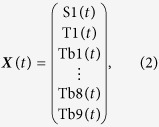


***A*** is the matrix of constants that characterizes the relationship between dynamics of each species and other species in Tb_9_ cluster and is given in the following equation:


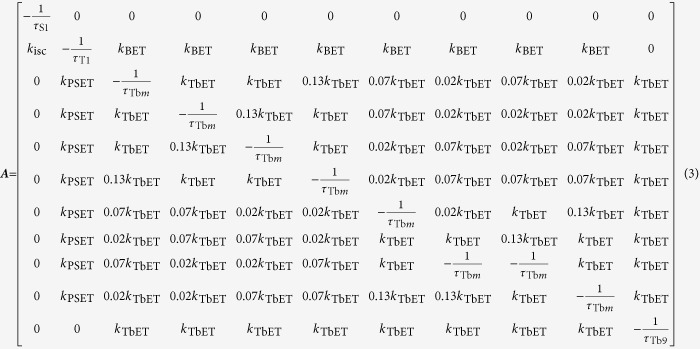


where


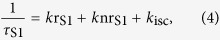



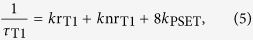



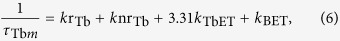



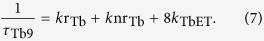


*k*_isc_, *k*r, *k*nr, *k*_PSET_, *k*_BET_, and *k*_TbET_ are defined as intersystem-crossing, radiative, nonradiative, PSET, BET, and TbET rate constants, respectively. The subscript indicates a species in a Tb_9_ cluster. Finally, vector ***J***(*t*) represents the input function of each species in Tb_9_ cluster:


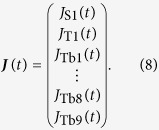


The excited state decay dynamics after short-pulse excitation were solved using Dirac’s delta function, and the quantum yield (steady-state excitation) was determined by using constants (Supplementary Information S3). In this calculation, the ground state population density is approximated as always being constant, and doubly excited Tb_9_ clusters are ignored because excitation with reasonably low pump intensity (under 20 W mm^−2^) is being considered^41^. The population density of S1(*t*) (excited singlet state) after excitation is normalized to unity.

**Figure 2:**
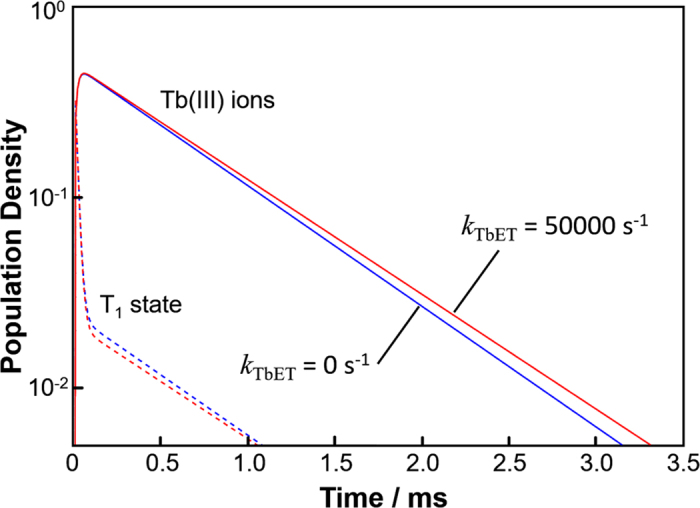
Calculated time evolution of population density of Tb_9_ cluster after short-pulse excitation of the ligands when TbET is present (*k*_TbET_ = 50000 s^−1^, red) and when TbET is absent (*k*_TbET_ = 0 s^−1^, blue). The solid and dashed lines represent the collective population density of Tb(III) ions and T_1_ state, respectively.

Figure 2 shows the calculated decay curve of the population density of Tb_9_ cluster following a short-pulse excitation for *k*_TbET_ = 0 s^−1^ (absence of TbET: blue line) or *k*_TbET_ = 50000 s^−1^ (presence of TbET: red line) in the presence of BET. The other rates *k***r**_S1_ + *k*n**r**_S1_ = 3.4 × 10^8^ s^−1^, *k*isc_s1_ = 7.5 × 10^8^ s^−1^, *k***r**_T1_ + *k*nr_T1_ = 15000 s^−1^, and *k*r_Tb_ = *k*nr_Tb_ = 400 s^−1^ were chosen from previous reports^53^,^54^. The values of *k*_PSET_ and *k*_BET_ are arbitrary and do not affect the qualitative result. For clarity of the results, *k*_PSET_ = 6000 s^−1^ and *k*_BET_ = 3000 s^−1^ were chosen. The lifetime of collective Tb(III) ions (Tb1(*t*) + Tb2(*t*) + … + Tb9(*t*)) is extended to *τ*_calc_ = 720 μs in the presence of TbET compared to *τ*_calc_ = 685 μs in the absence of TbET. The decay curves of the T_1_ state in Fig. 2 contain short (0–0.1 ms region) and long components (0.1 ms and onward). The short component corresponds to the relaxation process of the T_1_ state following intersystem crossing from the S_1_ state. The time of this process matches that of the rise in population density of Tb(III) ions. The longer component corresponds to the repopulation of the T_1_ state by BET from Tb(III) ions and as a result the decay rate is the same as for the Tb(III) ions. Population density of the longer component of the T_1_ state in the presence of TbET is lower than that in the absence of TbET, indicating suppression of BET. Table 1 summarizes the emission quantum yield *Φ*_ππ*,calc_ and BET efficiency *η*_BET,calc_ obtained by the calculation for steady-state excitation. BET efficiency *η*_BET,calc_ is defined as the difference in yield of excited Tb(III) ions with BET (*k*_BET_ = 3000 s^−1^) and without BET (*k*_BET_ = 0 s^−1^). An approximately 3% decrease in BET efficiency *η*_BET,calc_ is observed in the Tb_9_ cluster in the presence of TbET compared to that in the absence of TbET. We also calculated the population density for a hypothetical cluster where all Tb(III) ions are in an identical environment to confirm the importance of Tb9 being isolated from BET (Supplementary Information S5 and Table S3). In this case, no change was observed in the emission lifetime, quantum yield, or BET efficiency regardless of the rate of TbET. These calculations clearly show that the combination of fast TbET and Tb9 plays a role in the suppression of BET in Tb_9_ cluster.

### Structure and Identification

**Figure 3:**
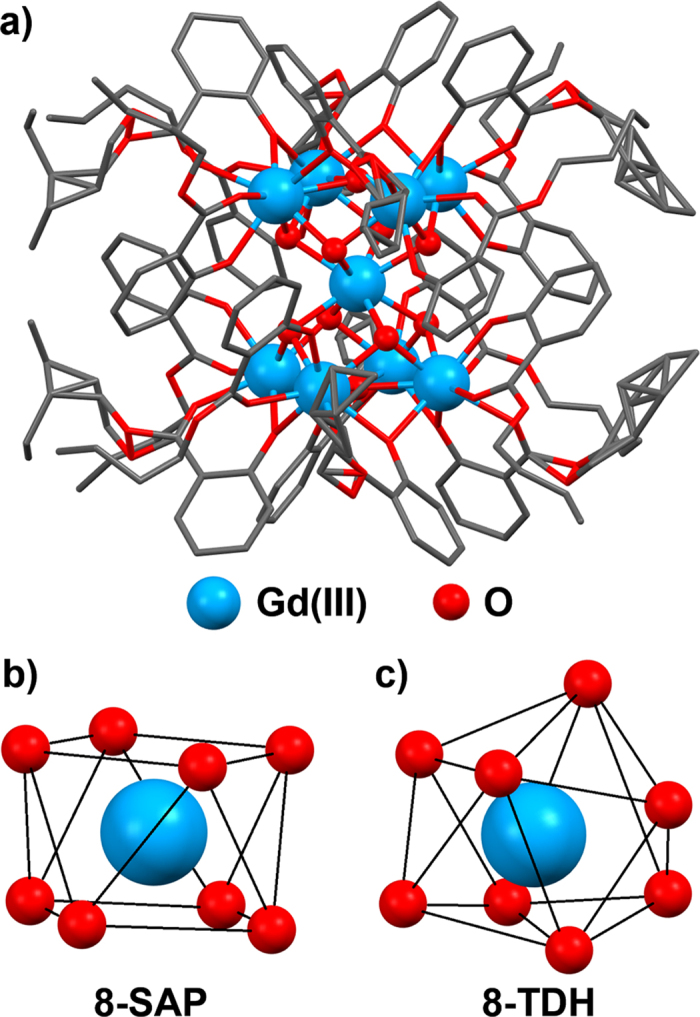
Molecular structure of Gd_9_ cluster. (**a**) Structure of Gd_9_ cluster (hydrogen atoms and NO_3_^−^ counter anion omitted for clarity), (**b**) Gd(III) ion with 8-SAP geometry, and (**c**) Gd(III) ion with 8-TDH geometry.

X-ray single-crystal analysis of Gd_9_ cluster was performed in order to understand the molecular structure and coordination geometry of the nine Ln(III) ions in Tb_n_Gd_9−n_ clusters. The analysis showed that Gd(III) ions in the nonanuclear cluster take the form of an “hour-glass” structure in which the upper four and lower four Gd(III) ions are connected to the center Gd(III) ion via oxygen-bridging as shown in Fig. 3a. All Gd (III) ions take the form of an 8-coordination structure. The center Gd(III) ion is coordinated only by eight oxygen atoms. Each of the Gd(III) ions in the upper four and lower four sites (outer unit) are coordinated by both butylsalicylate ligands and oxygen atoms. The distances between two Gd(III) ions for all combinations are summarized in Table S4.

Evaluation of coordination geometry of Gd(III) ions was done through continuous shape measures (CShM) calculation using SHAPE^55^,^56^,^57^. The CShM criterion *S*, summarized in Table S5, represents the degree of deviation from ideal coordination geometry. We have chosen CShM over another method called “shape measure” (ShM)^58^ because it takes account of the distortion of center metal ion from the center of mass. The principle and comparison of the calculation method in CShM and ShM is described in Supplementary Information S7. The *S* value for the center Gd(III) ion (Fig. 3b) was 0.082 when calculated for 8-coordinated square antiprism (8-SAP) geometry and it was 2.481 for 8-coordinated trigonal dodecahedron (8-TDH) geometry. Therefore, the coordination geometry of the center Gd(III) ion is attributed to the 8-SAP structure. The upper four and lower four Gd(III) ionns (Fig. 3c) take an 8-TDH structure since the *S* values calculated for 8-TDH are smaller than those for 8-SAP. This structural result is similar to that of our previously reported Tb_9_ cluster^50^.

The structures of the other Tb_n_Gd_9−n_ clusters (*n* = 1, 2, 5, 8) were determined by the combination of powder XRD (Figure S2a) and FAB-MS (Figure S2b). In the XRD results, the diffraction angle is corrected on the Si peak at 2*θ* = 28.43 degrees for accurate comparison of the peaks derived from the Tb_n_Gd_9−n_ clusters. Three distinguished peaks (2*θ*_1_ = 11.3 degrees, 2*θ*_2_ = 15.6 degrees, and 2*θ*_3_ = 19.8 degrees) were observed for all of the clusters indicating that the mixed Tb/Gd clusters have the same structure as those of Tb_9_ and Gd_9_ clusters. This is further supported by FAB-MS spectroscopy which showed m/z value of the Tb_n_Gd_9−n_ clusters corresponding to their calculated molecular weight without the NO_3_^−^ counter-anion. The results presented above indicate that all Tb_n_Gd_9−n_ clusters were successfully synthesized with identical structures.

### Optical Properties

**Figure 4:**
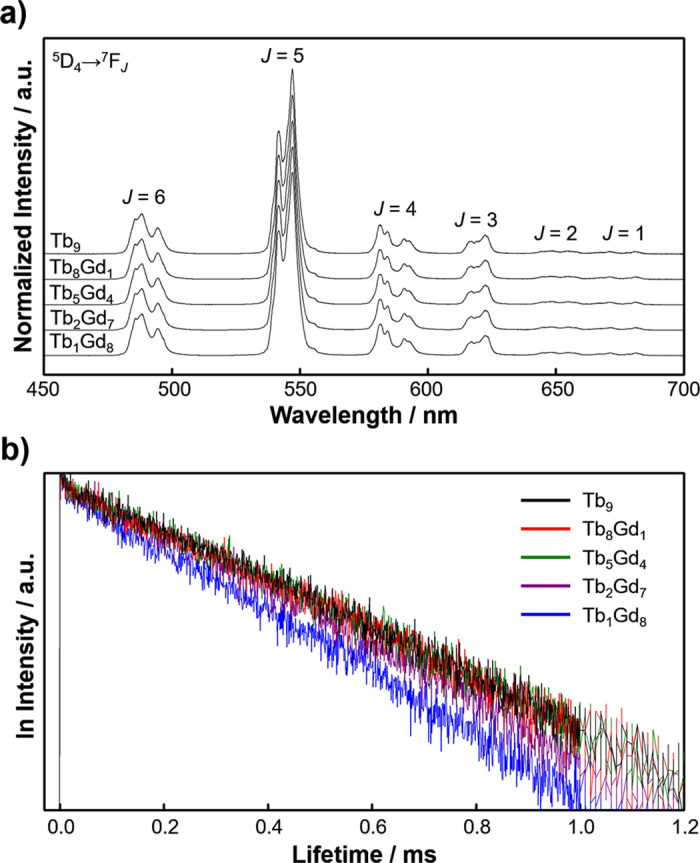
(**a**) Photoluminescence spectra of Tb_n_Gd_9−n_ clusters in 1.0 × 10^−4^ M chloroform solution normalized at the peak top. Excitation wavelength *λ*_EX_ was 380 nm. (**b**) Normalized emission lifetimes of Tb_n_Gd_9−n_ clusters in 1.0 × 10^−4^ M chloroform solution at 300 K. All spectra were normalized at emission intensity at 0 seconds. All samples were excited by nanosecond pulse laser at *λ*_EX_ = 355 nm and monitored at *λ*_EM_ = 550 nm (^5^D_4_ → ^7^F_6_ transition).

Emission spectra of Tb_n_Gd_9−n_ clusters (*n* = 1, 2, 5, 8, 9) in 1.0 × 10^−4^ M chloroform solution were measured and Fig. 4a shows the spectra, normalized at the peak intensity. The Tb_n_Gd_9−n_ clusters exhibit characteristic emission of the 4f-4f transition of the Tb(III) ion with each of the peaks corresponding to its ^5^D_4_ → ^7^F_*J*_ (*J* = 6 − 1) transitions^59^. The spectral shape of the emission was identical in all Tb_n_Gd_9−n_ clusters. Additionally, the emission spectral shape of Tb_9_ cluster in powder form (Figure S4) was the same as that in the solution form, indicating that the coordination structure is maintained in solution. The emission spectrum of Gd_9_ cluster in 1.0 × 10^−4^ M chloroform solution at 210 K (Figure S5) was measured to estimate the excited triplet state (T_1_) energy level of the ligand^38^,^60^,^61^. The emission peak was observed at 461 nm (21690 cm^−1^). Since the ^5^D_4_ level of a Tb(III) ion is 20620 cm^−1^ as observed in the emission spectra (485 nm), the energy gap *ΔE* between T_1_ and ^5^D_4_ was estimated to be 1070 cm^−1^. This is well within the range for BET in Tb(III) complexes (*ΔE* < 1850 cm^−1^)^33^,^34^,^38^, and thus BET is expected to take place in Tb_n_Gd_9−n_ clusters. The absolute emission quantum yield was determined by using an integration sphere (*λ*_EX_ = 380 nm, ligand excitation) and emission lifetimes were determined by using a nanosecond pulse laser (*λ*_EX_ = 355 nm, *λ*_EM_ = 550 nm, ligand excitation) for clusters in a chloroform solution. The emission decay profiles were single-exponential (Fig. 4b) in all Tb_n_Gd_9−n_ clusters. As summarized in Table 2, the emission quantum yield *Φ*_ππ*_ and lifetime *τ*_obs_ increased with increasing numbers of Tb(III) ions in the Tb_n_Gd_9−n_ clusters. The emission quantum yield reflects the effect of all photophysical processes in Tb_n_Gd_9−n_ clusters. Of these processes, TbET, PSET, and BET are all likely to vary with *n* in the Tb_n_Gd_9−n_ clusters, and the effect purely based on TbET is indistinguishable.

**Figure 5:**
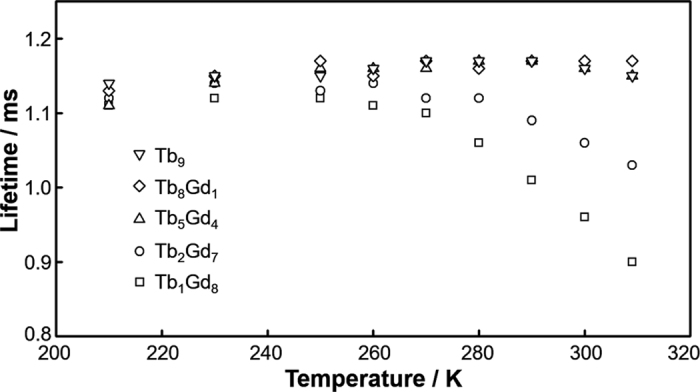
Emission lifetime temperature dependency of Tb_n_Gd_9−n_ clusters in 1.0 × 10^−4^ M chloroform solution. The experimental uncertainty in the reported lifetimes is up to ±1.5%.

The effect of TbET with increasing number of Tb(III) ions in the clusters was investigated by measuring the temperature dependence of emission lifetimes (Fig. 5). A clear temperature dependence was observed above 270 K for Tb_1_Gd_8_ and Tb_2_Gd_7_ clusters, while lifetimes remained constant for Tb_5_Gd_4_, Tb_8_Gd_1_, and Tb_9_ clusters. The rate constant of BET *k*_BET_ can be estimated by the following equation^37^,^62^.


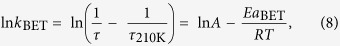


where *τ* is measured emission lifetime at a given temperature, *τ*_210K_ is the emission lifetime at 210 K, *A* is frequency factor, *Ea*_BET_ is activation energy for BET, *R* is gas constant, and *T* is temperature. *Ea*_BET_ and *k*_BET_ can be calculated for Tb_1_Gd_8_ cluster by an Arrhenius plot within the range in which temperature dependency was observed (Figure S6). The BET rate constant *k*_BET_ and activation energy *Ea*_BET_ for Tb_1_Gd_8_ cluster were calculated to be 167 s^−1^ and 38.7 kJ mol^−1^, respectively (Table 2).

Emission lifetime and its temperature dependence are in qualitative agreement with theoretical results presented in the Theoretical Background section. The theoretical lifetime of Tb_9_ cluster in the absence of BET is constant regardless of TbET rate *k*_TbET_ (Supplementary Information S4 and Table S2). Experimentally, BET is absent in Tb_n_Gd_9−n_ clusters at 210 K, and the number of Tb(III) ions in the cluster is directly related to *k*_TbET_. Thus the emission lifetime at this temperature should be the same for all Tb_n_Gd_9−n_ clusters. The experimental emission lifetimes of Tb_n_Gd_9−n_ clusters at 210 K *τ*_210K_ are approximately 1.12 ms regardless of the number of Tb(III) ions in the clusters (Table 2 and Fig. 5). Meanwhile, the theoretical lifetime of Tb_9_ clusters in the presence of BET shows that the lifetime is longer in the presence of TbET than in the absence of TbET. This comparison is analogous to experimental lifetimes of Tb_9_ cluster (presence of TbET) and Tb_1_Gd_8_ cluster (absence of TbET) at a temperature above 270 K, at which BET occurs. At a temperature above 270 K, the experimental lifetime is clearly longer for clusters with larger numbers of Tb(III) ions than for clusters with smaller numbers of Tb(III) ions. Such agreement between the theoretical and experimental results implies that the contribution of BET is indeed suppressed in Tb_9_ cluster because of TbET and the existence of a center Tb(III) ion not in direct contact with ligands for which no BET occurs.

### Summary and Conclusion

The role of energy transfer between Tb(III) ions in luminescent Tb(III) complexes has been demonstrated to reduce BET losses both theoretically and experimentally using Tb_n_Gd_9−n_ clusters ([Tb_n_Gd_9−n_(μ-OH)_10_(butylsalicylate)_16_]^+^NO_3_^−^) as model system. In the Theoretical Background section, we demonstrated that two features of Tb_9_ cluster were important for suppressing BET: 1) TbET by closely assembled Tb(III) ions and 2) existence of Tb9 that is isolated from BET. Experimentally, we synthesized and investigated the photophysical properties of nonanuclear Tb_n_Gd_9−n_ clusters where *n* = 0, 1, 2, 5, 8, and 9. The combination of X-ray single-crystal analysis, powder XRD, and FAB-MS revealed that these clusters have nearly identical structures. Temperature dependency measurements of emission lifetime revealed that effect of BET becomes prominent in Tb_n_Gd_9−n_ clusters at temperatures above 270 K. Below this temperature, the emission lifetime is constant for all clusters. Above 270 K, the decrease in emission lifetime is mitigated for clusters with over five Tb(III) ions. By comparing the trends observed in experimental results to those of theoretical results, it was found that the contribution of BET is indeed suppressed in clusters with a large number of Tb(III) ions. These findings provide a new insight into the fundamental photophysics of Ln(III) complexes as well as indication of a novel strategy to achieve higher luminescence efficiency in Ln(III) complexes.

## Methods

### Materials

Terbium(III) nitrate hexahydrate (Tb(NO_3_)_3_·6H_2_O) and triethylamine (C_6_H_15_N) were purchased from Kanto Chemical Co. Inc. Gadolinium(III) nitrate hexahydrate (Gd(NO_3_)_3_·6H_2_O) was purchased from Sigma-Aldrich Co. Butylsalicylate (C_11_H_14_O_3_) was purchased from Tokyo Chemical Industry Co., LTD. All other chemicals and solvents were of reagent grade and were used without further purification.

### Apparatus

FAB-MS spectra were measured on a JEOL JMS-700TZ. Elemental analyses were performed by Exter Analytical CE440. Infrared spectra were recorded on a JASCO FT/IR-4600 spectrometer. XRD spectra were characterized by a RIGAKU X-ray diffractometer RINT 2200. Single crystal X-ray diffractions were made on a RIGAKU RAXIS RAPID imaging plate area detector.

### Optical Measurements

Absorption spectra of Tb_n_Gd_9−n_ clusters were obtained by using a JASCO V-670 spectrometer. Emission spectra were measured using a Horiba/Jobin-Yvon FluoroLog-3 spectrofluorometer and a JASCO FP-6600 spectrometer. The combination of an integration sphere and a JASCO FP-6600 spectrometer was used to measure emission quantum yields. Emission lifetimes were measured using the third harmonic (355 nm) of a Q-switched Nd:YAG laser. The temperature was controlled using an Oxford Instruments OptistatDN2 cryostat.

### Crystallography

Colorless single crystals of Gd_9_ cluster obtained from solutions in methanol were mounted on a glass fiber by using epoxy resin glue. All measurements were made using a Rigaku RAXIS RAPID imaging plate area detector with graphite-monochromated MoKα radiation. Corrections for decay and Lorentz-polarization effects were made using a spherical absorption correction, solved by direct methods, and expanded using Fourier techniques. Non-hydrogen atoms were refined anisotropically except for disordered atoms. Hydrogen atoms were refined using the riding model. The final cycle of full-matrix least-squares refinement was based on observed reflections and variable parameters. All calculations were performed using a CrystalStructure crystallographic software package. We confirmed the CIF data by using the checkCIF/PLATON service. CCDC-1479981 (Gd_9_ cluster) contains the crystallographic data for this paper. These data can be obtained free of charge from The Cambridge Crystallographic Data Center via www.ccdc.cam.ac.uk/data_request/cif.

### Synthesis of Gd_9_ cluster ([Gd_9_(μ-OH)_10_(butylsalicylate)_16_]^+^NO_3_^−^)

Butylsalicylate (1.05 g, 5.40 mmol) was dissolved in methanol, and triethylamine (1.22 ml, 8.80 mmol) was added to the solution and the solution was stirred at 40 °C. Then Gd(NO_3_)_3_·6H_2_O (1.372 g, 3.04 mmol) in methanol was added dropwise to the solution and the solution stirred for 30 minutes. The solution was cooled to room temperature, and white powder Gd_9_ cluster ([Gd_9_(μ-OH)_10_(butylsalicylate)_16_]^+^NO_3_^−^) was obtained 15 minutes later. Yield: 59%. Selected IR (ATR, cm^−1^): 3573 (w, O–H), 3234 (w, O–H), 2956 (m, C–H), 1318 (s, C–O). FAB-MS: m/z = 4676.8 [Gd_9_(μ-OH)_10_(butylsalicylate)_16_]^+^. Elemental analysis calculated for C_176_H_218_NO_61_Gd_9_: C, 44.61%, H, 4.64%, N, 0.30%. Found: C, 44.30%, H, 4.53%, N, 0.25%.

### Synthesis of Tb_1_Gd_8_ cluster ([Tb_1_Gd_8_(μ-OH)_10_(butylsalicylate)_16_]^+^NO_3_^−^)

Tb_1_Gd_8_ cluster was synthesized by the same procedure as that used for Gd_9_ cluster except for the use of Tb(NO_3_)_3_·6H_2_O (0.172 g, 0.380 mmol) and Gd(NO_3_)_3_·6H_2_O (1.24 g, 2.73 mmol). Yield: 82%. Selected IR (ATR, cm^−1^): 3573 (w, O–H), 3234 (w, O–H), 2956 (m, C–H), 1318 (s, C–O). FAB-MS: m/z = 4677.8 [Tb_1_Gd_8_(μ-OH)_10_(butylsalicylate)_16_]^+^. Elemental analysis calculated for C_176_H_218_NO_61_Tb_1_Gd_8_: C, 44.59%, H, 4.64%, N, 0.30%. Found: C, 43.95%, H, 4.53%, N, 0.29%.

### Synthesis of Tb_2_Gd_7_ cluster ([Tb_2_Gd_7_(μ-OH)_10_(butylsalicylate)_16_]^+^NO_3_^−^)

Tb_2_Gd_7_ cluster was synthesized by the same procedure as that used for Gd_9_ cluster except for the use of Tb(NO_3_)_3_·6H_2_O (0.306 g, 0.675 mmol) and Gd(NO_3_)_3_·6H_2_O (1.07 g, 2.36 mmol). Yield: 51%. Selected IR (ATR, cm^−1^): 3573 (w, O–H), 3234 (w, O–H), 2956 (m, C–H), 1318 (s, C–O). FAB-MS: m/z = 4679.8 [Tb_2_Gd_7_(μ-OH)_10_(butylsalicylate)_16_]^+^. Elemental analysis calculated for C_176_H_218_NO_61_Tb_2_Gd_7_: C, 44.58%, H, 4.63%, N, 0.30%. Found: C, 44.32%, H, 4.54%, N, 0.26%.

### Synthesis of Tb_5_Gd_4_ cluster ([Tb_5_Gd_4_(μ-OH)_10_(butylsalicylate)_16_]^+^NO_3_^−^)

Tb_5_Gd_4_ cluster was synthesized by the same procedure as that used for Gd_9_ cluster except for the use of Tb(NO_3_)_3_·6H_2_O (0.688 g, 1.52 mmol) and Gd(NO_3_)_3_·6H_2_O (0.684 g, 1.52 mmol). Yield: 41%. Selected IR (ATR, cm^−1^): 3573 (w, O–H), 3234 (w, O–H), 2956 (m, C–H), 1318 (s, C–O). FAB-MS: m/z = 4684.9 [Tb_5_Gd_4_(μ-OH)_10_(butylsalicylate)_16_]^+^. Elemental analysis calculated for C_176_H_218_NO_61_Tb_5_Gd_4_: C, 44.53%, H, 4.63%, N, 0.30%. Found: C, 44.23%, H, 4.52%, N, 0.24%.

### Synthesis of Tb_8_Gd_1_ cluster ([Tb_8_Gd_1_(μ-OH)_10_(butylsalicylate)_16_]^+^NO_3_^−^)

Tb_8_Gd_1_ cluster was synthesized by the same procedure as that used for Gd_9_ cluster except for the use of Tb(NO_3_)_3_·6H_2_O (1.026 g, 2.26 mmol) and Gd(NO_3_)_3_·6H_2_O (0.344 g, 0.762 mmol). Yield: 49%. Selected IR (ATR, cm^−1^): 3573 (w, O–H), 3234 (w, O–H), 2956 (m, C–H), 1318 (s, C–O). FAB-MS: m/z = 4689.8 [Tb_8_Gd_1_(μ-OH)_10_(butylsalicylate)_16_]^+^. Elemental analysis calculated for C_176_H_218_NO_61_Tb_8_Gd_1_: C, 44.48%, H, 4.62%, N, 0.29%. Found: C, 44.24%, H, 4.53%, N, 0.24%.

### Synthesis of Tb_9_ cluster ([Tb_9_(μ-OH)_10_(butylsalicylate)_16_]^+^NO_3_^−^)

Tb_9_ cluster was synthesized by the same procedure as that used for Gd_9_ cluster except for the use of Tb(NO_3_)_3_·6H_2_O (1.372 g, 3.04 mmol). Yield: 52%. Selected IR (ATR, cm^−1^): 3573 (w, O–H), 3234 (w, O–H), 2956 (m, C–H), 1318 (s, C–O). FAB-MS: m/z = 4691.3 [Tb_9_(μ-OH)_10_(butylsalicylate)_16_]^+^. Elemental analysis calculated for C_176_H_218_NO_61_Tb_9_: C, 44.47%, H, 4.62%, N, 0.29%. Found: C, 44.20%, H, 4.57%, N, 0.27%.

## Additional Information

**How to cite this article**: Omagari, S. *et al*. Critical Role of Energy Transfer Between Terbium Ions for Suppression of Back Energy Transfer in Nonanuclear Terbium Clusters. *Sci. Rep.* **6**, 37008; doi: 10.1038/srep37008 (2016).

**Publisher’s note**: Springer Nature remains neutral with regard to jurisdictional claims in published maps and institutional affiliations.

## Supplementary Material

Supplementary Information

## Figures and Tables

**Table 1:** Calculated lifetimes, quantum yields and BET efficiencies of Tb_9_ cluster.

***k***_**TbET**_/**s**^**−1**^	***τ***_**calc**_**/μs**	***Φ***_ππ*,calc_	***η***_**BET,calc**_
50000 (presence of TbET)	720	14.1%	44.3%
0 (absence of TbET)	685	13.4%	47.2%

**Table 2:** Photophysical properties of Tb_n_Gd_9−n_ clusters.

Clusters	***Φ***_ππ*,_^a^	***τ***_**obs**_**/ms**^b^	***τ***_**210K**_**/ms**^b^	***A*****/10**^**8**^** s**^**−1**^^c^	***k***_**BET**_**/s**^**−1**^^c^	***Ea***_**BET**_**/kj mol**^**−1**^^d^
Tb_1_Gd_8_	14%	0.96	1.11	9.10	167	38.7
Tb_2_Gd_7_	23%	1.06	1.12	*	*	*
Tb_5_Gd_4_	33%	1.16	1.11	*	*	*
Tb_8_Gd_1_	40%	1.17	1.13	*	*	*
Tb_9_	39%	1.16	1.14	*	*	*

^a^Measured in1.0 × 10^−4^ M chloroform solution (*λ*_EX_ = 380 nm).

^b^Measured in 1.0 × 10^−4^ M chloroform solution (*λ*_EX_ = 355 nm).

^c^Calculated from Equation (9). *A* is frequency factor.

^d^Analyzed from an Arrhenius plot of Equation (9) using lifetime temperature dependency results. *k*_BET_ and *E*_*a*BET_ could only be calculated for Tb_1_Gd_8_ cluster since other Tb_n_Gd_9−n_ clusters (*n* = 2, 5, 8, 9) involve TbET, which contributes to the temperature dependency of lifetimes.
