# Supplementary material for: Critical Role of Energy Transfer Between Terbium Ions for Suppression of Back Energy Transfer in Nonanuclear Terbium Clusters
Source: Sci Rep. 2016 Nov 15;6:37008. doi: 10.1038/srep37008 (PMC5109476; doi:10.1038/srep37008)
Supplement: Supplementary Information [file srep37008-s1.pdf]

SUPPLEMENTARY INFORMATION

Critical Role of Energy Transfer Between Terbium Ions for Suppression of Back Energy Transfer in Nonanuclear Terbium Clusters

Shun Omagari,[1] Takayuki Nakanishi,[2]* Yuichi Kitagawa,[2] Tomohiro Seki,[2] Koji Fushimi,[2] Hajime Ito,[2] Andries Meijerink,[3] and Yasuchika Hasegawa[2]*

[1]Graduate School of Chemical Sciences and Engineering, Hokkaido University, N13 W8, Kita-ku, Sapporo, Hokkaido 060–8628, Japan
[2]Faculty of Engineering, Hokkaido University, N13 W8, Kita-ku, Sapporo, Hokkaido 060–8628, Japan
[3]Debye Institute, Department of Chemistry, Utrecht University, Princetonplein 5, 3584 CC Utrecht, The Netherlands
*nakanishi@eng.hokudai.ac.jp, hasegaway@eng.hokudai.ac.jp

Energy Transfer and distance dependency

Energy transfer is dominated by multipole and exchange interaction. For multipole interactions, dipole-dipole (Förster resonance energy transfer[1]), dipole-quadrupole, and quadrupole-quadrupole terms are relevant. The energy transfer rate constants for these terms (W_{dd}, W_{dq}, and W_{qq}) are given by the following expression for two-atom system[2–4]:

$$W_{\mathrm{dd}} = \frac{2\pi}{\hbar}\frac{2}{3}\frac{e^4}{R^6}|\langle \mathrm{A}'|\boldsymbol{D}^1|\mathrm{A}\rangle|^2|\langle \mathrm{B}|\boldsymbol{D}^1|\mathrm{B}'\rangle|^2 F\,, \tag{10}$$

$$W_{\mathrm{dq}} = \frac{2\pi}{\hbar}\frac{e^4}{R^8}\left[|\langle \mathrm{A}'|\boldsymbol{D}^1|\mathrm{A}\rangle|^2|\langle \mathrm{B}|\boldsymbol{D}^2|\mathrm{B}'\rangle|^2 + |\langle \mathrm{A}'|\boldsymbol{D}^2|\mathrm{A}\rangle|^2|\langle \mathrm{B}|\boldsymbol{D}^1|\mathrm{B}'\rangle|^2\right] F\,, \tag{11}$$

$$W_{\mathrm{qq}} = \frac{2\pi}{\hbar}\frac{14}{5}\frac{e^4}{R^{10}}|\langle \mathrm{A}'|\boldsymbol{D}^2|\mathrm{A}\rangle|^2|\langle \mathrm{B}|\boldsymbol{D}^2|\mathrm{B}'\rangle|^2 F\,, \tag{12}$$

where R is the distance between a donor (A) and an acceptor (B), F is the sum over Franck-Condon factors and spectral overlap, the matrix element $\langle \mathrm{X}'|\boldsymbol{D}^l|\mathrm{X}\rangle$ is the transition dipole (l = 1) or quadrupole (l = 2) moment of the transition from state X' to X. For the exchange interaction W_{ex}[3–5],

$$W_{\mathrm{ex}} = \frac{2\pi}{\hbar}|\langle 4f_{\mathrm{A}}|4f_{\mathrm{B}}\rangle|^4\left|\left\langle \mathrm{AB}'\middle|\sum_{ij}\left(\frac{1}{2}+2\vec{s}_i\cdot\vec{s}_j\right)\frac{e^2}{r_{ij}}\middle|\mathrm{A}'\mathrm{B}\right\rangle\right|^2 F\,, \tag{13}$$

where $\langle 4f_{\mathrm{A}}|4f_{\mathrm{B}}\rangle^2$ is the Mülliken-type approximated radial overlap integral and $\left\langle \mathrm{AB}'\middle|\sum_{ij}\left(\frac{1}{2}+2\vec{s}_i\cdot\vec{s}_j\right)\frac{e^2}{r_{ij}}\middle|\mathrm{A}'\mathrm{B}\right\rangle$ is the exchange integral. The energy transfer rate when considering only the exchange integral is often approximated in a well-known Dexter's formulation as,

$$W_{\mathrm{Dexter}} = k_0 e^{-\frac{2R}{L}}\,, \tag{14}$$

where k_0 is a constant consisting of normalized spectral overlap integral and orbital interactions, and L is effective Bohr radius.

Equations (10) – (12) indicate that dipole-dipole, dipole-quadrupole, and quadrupole-quadrupole interactions have the donor-acceptor distance dependence of 6th power, 8th power, and 10th power, respectively. Meanwhile, equation (13) shows that the exchange interactions depends on radial overlap integral and exchange integral. Since radial overlap integral is also distance dependent, the distance dependence of W_{ex} goes beyond single-exponential.

In Tb_9 clusters, the closest Tb(III)-Tb(III) pairs are separated by 3.65 Å. At this distance, all multipole interaction may have contributions that cannot be ignored. For instance, Malta reported that theoretically, quadrupole-quadrupole interaction is the main mechanism of energy transfer in the distance below 4 Å in an Yb(III)-Yb(III) pair, and exchange interactions also have some contribution. This may also be the case for Tb(III)-Tb(III) pair, although the energy transfer rate is expected to be smaller than Yb(III)-Yb(III) pair since oscillator strength of $^7F_6 \rightarrow {}^5D_4$ transition of Tb(III) is usually two orders of magnitude smaller than $^7F_{7/2} \rightarrow {}^7F_{5/2}$ transition of Yb(III).[6,7] In such case, any other pairs than the closest Tb(III)-Tb(III) pairs may have relatively much smaller energy transfer rate.

In the Theoretical Background section, for the sake of discussion and for simplicity, only the dipole-dipole interaction was considered. However, from the above considerations, we have also calculated a case where TbET only occur for the closest Tb(III)-Tb(III) pairs. This means that k_{TbET} = 0 s^{-1} for any other pairs. The results are shown in Table S1. These values are almost identical to the values calculated in the Theoretical Background section with the difference of less than 0.01%. This indicates that the type of interaction in Tb_9 cluster does not significantly affect the outcome.

Table S1. Calculated lifetimes, quantum yields and BET efficiencies of Tb_9 cluster with TbEnT only considered for the closest pair.

k_{TbET} / s^{-1}	τ_{calc} / μs	$\Phi_{\pi\pi*,\mathrm{calc}}$	$\eta_{\mathrm{BET,calc}}$
50000 (presence of TbET)	720	14.1%	44.3%
0 (absence of TbET)	685	13.4%	47.2%

Rate equation with different input function

This section presents procedures for obtaining an appropriate rate equation for Tb_9 clusters with different excitation method. Equation (1) is considered as an inhomogeneous system of linear ordinary differential equation in the matrix form. An analytical solution of Equation (1) with general input function $\boldsymbol{J}(t)$ when initial value $\boldsymbol{X}(0) = \boldsymbol{0}$ is:

$$\boldsymbol{X}(\mathrm{t}) = \int_0^t \mathrm{e}^{\boldsymbol{A}(t-t')}\boldsymbol{J}(t')\mathrm{d}t' \quad . \tag{15}$$

The final result was obtained after input of numerical values to the coefficients. Since direct calculation of matrix exponential $\mathrm{e}^{\boldsymbol{A}t}$ is time consuming and sometimes unsolvable analytically, diagonalization was performed. MATLAB was used to solve the system of differential equations.

Short-pulse ligand excitation. For short-pulse ligand excitation of Tb_9 cluster, the input function consists of Dirac's delta function $\delta(t')$ for the excited singlet state (S_1 specie) and 0 for other species. The input function $\boldsymbol{J}(t)$ (Equation (8)) becomes:

$$\boldsymbol{J}(t) = \begin{pmatrix} \delta(t') \\ 0 \\ \vdots \\ 0 \end{pmatrix} . \tag{16}$$

With this input function, Equation (15) becomes:

$$\boldsymbol{X}(\mathrm{t}) = \mathrm{e}^{\boldsymbol{A}t}\boldsymbol{B} \quad , \tag{17}$$

$$\text{where} \quad \boldsymbol{B} = \begin{pmatrix} 1 \\ 0 \\ \vdots \\ 0 \end{pmatrix} .$$

This gives population density decay of nonanuclear Tb(III) cluster normalized at 1.

Steady-state excitation. For steady state ligand excitation of nonanuclear Tb(III) cluster, the input function consists of a constant for excited singlet state (S_1 specie) and 0 for other species. The input function becomes:

$$\boldsymbol{J}(t) = \begin{pmatrix} 1 \\ 0 \\ \vdots \\ 0 \end{pmatrix} . \tag{18}$$

With this input function, Equation (15) becomes:

$$\boldsymbol{X}(t) = \boldsymbol{A}^{-1}\,(\mathrm{e}^{-\boldsymbol{A}t} - \boldsymbol{E})\boldsymbol{B} = \boldsymbol{A}^{-1}(\mathrm{e}^{\boldsymbol{A}t} - \boldsymbol{E})\boldsymbol{B} \quad , \tag{19}$$

$$\text{where} \quad \boldsymbol{B} = \begin{pmatrix} 1 \\ 0 \\ \vdots \\ 0 \end{pmatrix} .$$

$\boldsymbol{E}$ is an identity matrix. By multiplying the radiative rate constant $k\mathrm{r}$ to a specific element of $\boldsymbol{X}(t)$ (specie of Tb_9 cluster), quantum yield of the specie can be obtained. In the case of quantum yield of Tb(III) ion emission, this would be:

$$\Phi_{\pi\pi*\mathrm{calc}} = k\mathrm{r}_{\mathrm{Tb}}(\mathrm{Tb1}(t) + \mathrm{Tb2}(t) + \cdots + \mathrm{Tb8}(t) + \mathrm{Tb9}(t)) \quad . \tag{20}$$

BET efficiency $\eta_{\mathrm{BET,calc}}$ is defined by the percentage of loss of population density of Tb(III) ions in the presence of BET to when BET is absent.

$$\eta_{\mathrm{BET}} = \frac{[\mathrm{Tb1}(t) + \mathrm{Tb2}(t) + \cdots + \mathrm{Tb8}(t) + \mathrm{Tb9}(t)]_{k_{\mathrm{BET}}=0} - [\mathrm{Tb1}(t) + \mathrm{Tb2}(t) + \cdots + \mathrm{Tb8}(t) + \mathrm{Tb9}(t)]_{k_{\mathrm{BET}}=3000}}{[\mathrm{Tb1}(t) + \mathrm{Tb2}(t) + \cdots + \mathrm{Tb8}(t) + \mathrm{Tb9}(t)]_{k_{\mathrm{BET}}=0}} \quad . \tag{21}$$

Although Equation (19) is time dependent (and therefore Equation (20) and (21) as well), when sufficient time (in the order of few seconds) has elapsed from initiation of steady-excitation, it converges to a certain value and becomes time independent.

TbET rate constant dependency in absence of BET

TbET rate constant dependency on the lifetime of Tb_9 cluster in the absence of BET was evaluated by the same method presented in the Theoretical Background section, but with different values for TbET rate constant k_{TbET} (0, 500, 5000, and 50000 s^{-1}). BET rate constant k_{BET} is 0 s^{-1}, representing the absence of BET. All other rate constants $k\mathrm{r}_{\mathrm{S1}}$ + $k\mathrm{nr}_{\mathrm{S1}}$, $k\mathrm{isc}_{\mathrm{S1}}$, $k\mathrm{r}_{\mathrm{T1}}$ + $k\mathrm{nr}_{\mathrm{T1}}$, k_{PSET}, $k\mathrm{r}_{\mathrm{Tb}}$, and $k\mathrm{nr}_{\mathrm{Tb}}$ are the same as those used in the Theoretical Background section. Calculated lifetime τ_{calc}, quantum yield $\Phi_{\pi\pi*,\mathrm{calc}}$, and BET efficiency $\eta_{\mathrm{BET,calc}}$ are summarized in Table S1. No dependency of TbET rate constant on the lifetime and quantum yield of Tb_9 cluster was observed, indicating that TbET serves no role in changing the photophysical properties of Tb_9 cluster in the absence of BET.

Table S2. TbET rate constant dependency on theoretical lifetime in absence of BET.

k_{TbET} / s^{-1}	τ_{calc} / μs	$\Phi_{\pi\pi*,\mathrm{calc}}$	$\eta_{\mathrm{BET,calc}}$
0	1300	25.28%	0%
500	1300	25.28%	0%
5000	1300	25.28%	0%
50000	1300	25.28%	0%

Population density decay and quantum yield of hypothetical cluster

The hypothetical cluster is a nonanuclear Tb(III) cluster where Tb9 is also involved in BET with the same rate as in Tb*m* (m = 1 – 8). Comparison with theoretical population density decay and quantum yield of Tb_9 cluster shows that the existence of Tb9 (BET does not efficiently take place) is important. The rate Equation (1) was solved with coefficient matrix $\boldsymbol{A}_\mathrm{h}$:

$$\boldsymbol{A}_\mathrm{h} = \begin{bmatrix}
-\frac{1}{\tau_{\mathrm{S1}}} & 0 & 0 & 0 & 0 & 0 & 0 & 0 & 0 & 0 & 0 \\
k_{\mathrm{isc}} & -\frac{1}{\tau_{\mathrm{T1}}} & k_{\mathrm{BET}} & k_{\mathrm{BET}} & k_{\mathrm{BET}} & k_{\mathrm{BET}} & k_{\mathrm{BET}} & k_{\mathrm{BET}} & k_{\mathrm{BET}} & k_{\mathrm{BET}} & k_{\mathrm{BET}} \\
0 & k_{\mathrm{PSET}} & -\frac{1}{\tau_{\mathrm{Tb}m}} & k_{\mathrm{TbET}} & k_{\mathrm{TbET}} & 0.13k_{\mathrm{TbET}} & 0.07k_{\mathrm{TbET}} & 0.02k_{\mathrm{TbET}} & 0.07k_{\mathrm{TbET}} & 0.02k_{\mathrm{TbET}} & k_{\mathrm{TbET}} \\
0 & k_{\mathrm{PSET}} & k_{\mathrm{TbET}} & -\frac{1}{\tau_{\mathrm{Tb}m}} & 0.13k_{\mathrm{TbET}} & k_{\mathrm{TbET}} & 0.07k_{\mathrm{TbET}} & 0.07k_{\mathrm{TbET}} & 0.02k_{\mathrm{TbET}} & 0.02k_{\mathrm{TbET}} & k_{\mathrm{TbET}} \\
0 & k_{\mathrm{PSET}} & k_{\mathrm{TbET}} & 0.13k_{\mathrm{TbET}} & -\frac{1}{\tau_{\mathrm{Tb}m}} & k_{\mathrm{TbET}} & 0.02k_{\mathrm{TbET}} & 0.07k_{\mathrm{TbET}} & 0.02k_{\mathrm{TbET}} & 0.07k_{\mathrm{TbET}} & k_{\mathrm{TbET}} \\
0 & k_{\mathrm{PSET}} & 0.13k_{\mathrm{TbET}} & k_{\mathrm{TbET}} & k_{\mathrm{TbET}} & -\frac{1}{\tau_{\mathrm{Tb}m}} & 0.02k_{\mathrm{TbET}} & 0.02k_{\mathrm{TbET}} & 0.07k_{\mathrm{TbET}} & 0.07k_{\mathrm{TbET}} & k_{\mathrm{TbET}} \\
0 & k_{\mathrm{PSET}} & 0.07k_{\mathrm{TbET}} & 0.07k_{\mathrm{TbET}} & 0.02k_{\mathrm{TbET}} & 0.02k_{\mathrm{TbET}} & -\frac{1}{\tau_{\mathrm{Tb}m}} & k_{\mathrm{TbET}} & k_{\mathrm{TbET}} & 0.13k_{\mathrm{TbET}} & k_{\mathrm{TbET}} \\
0 & k_{\mathrm{PSET}} & 0.02k_{\mathrm{TbET}} & 0.07k_{\mathrm{TbET}} & 0.07k_{\mathrm{TbET}} & 0.02k_{\mathrm{TbET}} & k_{\mathrm{TbET}} & -\frac{1}{\tau_{\mathrm{Tb}m}} & 0.13k_{\mathrm{TbET}} & k_{\mathrm{TbET}} & k_{\mathrm{TbET}} \\
0 & k_{\mathrm{PSET}} & 0.07k_{\mathrm{TbET}} & 0.02k_{\mathrm{TbET}} & 0.02k_{\mathrm{TbET}} & 0.07k_{\mathrm{TbET}} & k_{\mathrm{TbET}} & 0.13k_{\mathrm{TbET}} & -\frac{1}{\tau_{\mathrm{Tb}m}} & k_{\mathrm{TbET}} & k_{\mathrm{TbET}} \\
0 & k_{\mathrm{PSET}} & 0.02k_{\mathrm{TbET}} & 0.02k_{\mathrm{TbET}} & 0.07k_{\mathrm{TbET}} & 0.07k_{\mathrm{TbET}} & 0.13k_{\mathrm{TbET}} & k_{\mathrm{TbET}} & k_{\mathrm{TbET}} & -\frac{1}{\tau_{\mathrm{Tb}m}} & k_{\mathrm{TbET}} \\
0 & k_{\mathrm{PSET}} & k_{\mathrm{TbET}} & k_{\mathrm{TbET}} & k_{\mathrm{TbET}} & k_{\mathrm{TbET}} & k_{\mathrm{TbET}} & k_{\mathrm{TbET}} & k_{\mathrm{TbET}} & k_{\mathrm{TbET}} & -\frac{1}{\tau_{\mathrm{Tb9}}}
\end{bmatrix} \quad (22)$$

Unlike Equation (3) (coefficient matrix $\boldsymbol{A}$), row 2 column 11 of coefficient matrix $\boldsymbol{A}_\mathrm{h}$ is k_{BET} and row 11 column 2 is k_{PSET} instead of 0. This gives almost fully identical quenching environment of Tb9 to Tb*m* (m = 1 – 8).

Table S3. Calculated quantum yield and BET efficiency of hypothetical cluster of nine Tb(III) ions in direct contact with ligands.

k_{TbET} / s^{-1}	τ_{calc} / μs	$\Phi_{\pi\pi*,\mathrm{calc}}$	$\eta_{\mathrm{BET,calc}}$
50000 (presence of TbET)	713	14.31%	44.90%
0 (absence of TbET)	713	14.31%	44.90%

ORTEP of Gd_9 cluster

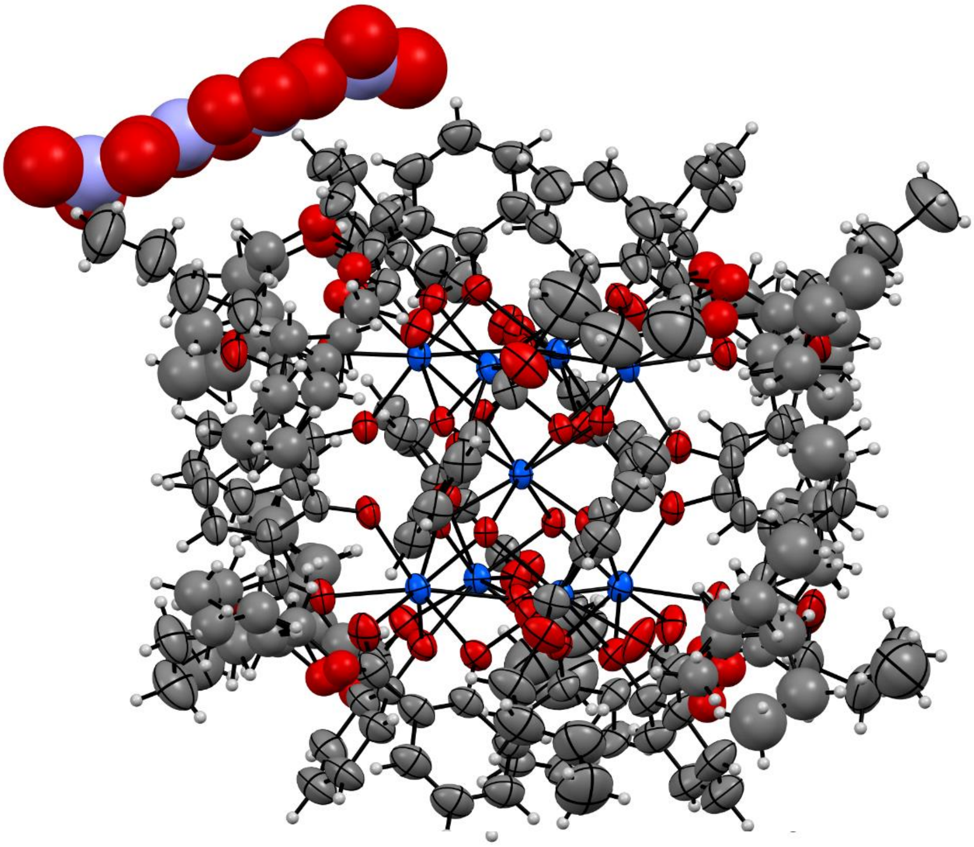

Figure S1. ORTEP representation of Gd_9 cluster. Color legend: gadolinium (blue), oxygen (red), carbon (grey), nitrogen (purple), hydrogen (white). Thermal ellipsoids are represented in 50% probability.

Table S4. Distance between Gd(III) ions for all combinations.

Combination*	Distance / Å	Combination*	Distance / Å	Combination*	Distance / Å
Gd1-Gd2	3.631	Gd1-Gd4	5.153	Gd4-Gd5	7.203
Gd2-Gd4	3.638	Gd2-Gd3	5.128	Gd4-Gd7	7.086
Gd4-Gd3	3.631	Gd5-Gd8	5.128	Gd3-Gd5	7.104
Gd3-Gd1	3.640	Gd6-Gd7	5.153	Gd3-Gd6	7.203
Gd5-Gd6	3.631	Gd1-Gd5	5.618		
Gd6-Gd8	3.638	Gd1-Gd7	5.717		
Gd8-Gd7	3.631	Gd2-Gd5	5.749		
Gd7-Gd5	3.640	Gd2-Gd6	5.622		
Gd9-Gd1	3.698	Gd3-Gd7	5.618		
Gd9-Gd2	3.691	Gd3-Gd8	5.749		
Gd9-Gd3	3.710	Gd4-Gd6	5.764		
Gd9-Gd4	3.709	Gd4-Gd8	5.622		
Gd9-Gd5	3. 710	Gd1-Gd6	7.086		
Gd9-Gd6	3.709	Gd1-Gd8	7.168		
Gd9-Gd7	3.698	Gd2-Gd7	7.168		
Gd9-Gd8	3.691	Gd2-Gd8	7.070		

*The labels of the Gd ions in this table are based on the labels used in Theoretical Background section and not the labels in the CIF file for consistency with the main text.

Continuous Shape Measure (CShM) and Shape Measure

Continuous shape measure (CShM) calculates the deviation of the vertices of an actual structure to the vertices of an ideal structure. The CShM criterion S_{CShM} is given by the following equation:[8,9]

$$S_{\mathrm{CShM}} = \min \frac{\sum_k^N |\boldsymbol{Q}_k - \boldsymbol{P}_k|^2}{\sum_k^N |\boldsymbol{Q}_k - \boldsymbol{Q}_0|^2} \times 100 \quad , \tag{23}$$

where $\boldsymbol{Q}_k$ is the vertices of an actual structure, $\boldsymbol{Q}_0$ is the center of mass of an actual structure, $\boldsymbol{P}_k$ is the vertices of an ideal structure, and N is the number of vertices. In the case of Gd_9 cluster, for each Gd(III) ion, N equals 9 (Gd(III) ion and eight oxygen atoms). CShM calculation takes accounts of the deviation of the metal atom from the center of mass.

On the other hand, shape measure (ShM) calculates the deviation of the dihedral angles of an actual structure to the dihedral angles of an ideal structure. The ShM criterion S_{ShM} is given by the following equation:[10]

$$S_{\mathrm{ShM}} = \min \sqrt{\frac{1}{M} \sum_i^M (\delta_i - \theta_i)^2} \times 100 \quad , \tag{24}$$

where δ_i is the vertices of an actual structure, θ_i is the center of mass of an actual structure, and M is the number of dihedral angles. In the case of Gd_9 cluster, for each Gd(III) ion, M equals 18. Since dihedral angles are independent of the effect of the distortion of the metal ion, ShM calculations do not take into consideration of the distortion of the metal ion.

Table S4 summarizes the difference in S_{CShM} values when the metal ion is taken into consideration to those of when metal ion are not considered. The importance of considering the deviation of the metal atom from the center of mass of a structure lies in the fact that without such consideration, the S values were smaller. This indicates that when the metal ion is not considered, the actual structure is calculated to be closer to ideal. Therefore, inclusion of the metal ion is indeed important to derive more accurate result. It is essential to point out that the S_{CShM} value of Gd9 calculated with and without the metal ion was almost identical. This implies that the distortion of the structure of Gd9 was purely due to the position of the oxygen atoms.

Table S5. Calculated S_CShM values for all Gd(III) ions with and without including metal ion.

Gd(III) ion	S_{SAP} (without)	S_{SAP} (with)	S_{TDH} (without)	S_{TDH} (with)
Gd1	4.670	4.725	2.920	2.976
Gd2	4.532	4.588	2.646	2.704
Gd3	4.752	4.813	2.774	2.836
Gd4	4.746	4.822	2.435	2.513
Gd5	4.752	4.813	2.774	2.836
Gd6	4.746	4.822	2.435	2.513
Gd7	4.670	4.725	2.920	2.976
Gd8	4.532	4.588	2.646	2.704
Gd9	0.082	0.082	2.481	2.481

S_{SAP} and S_{TDH} are S values calculated for an 8-coordinated square antiprism and an 8-coordinated trigonal dodecahedron, respectively. Label in parentheses indicates whether metal ion has been taken into consideration or not. The labels (numbering) of Gd(III) ions are based on the labels used for Tb_9 cluster in the Theoretical Background section.

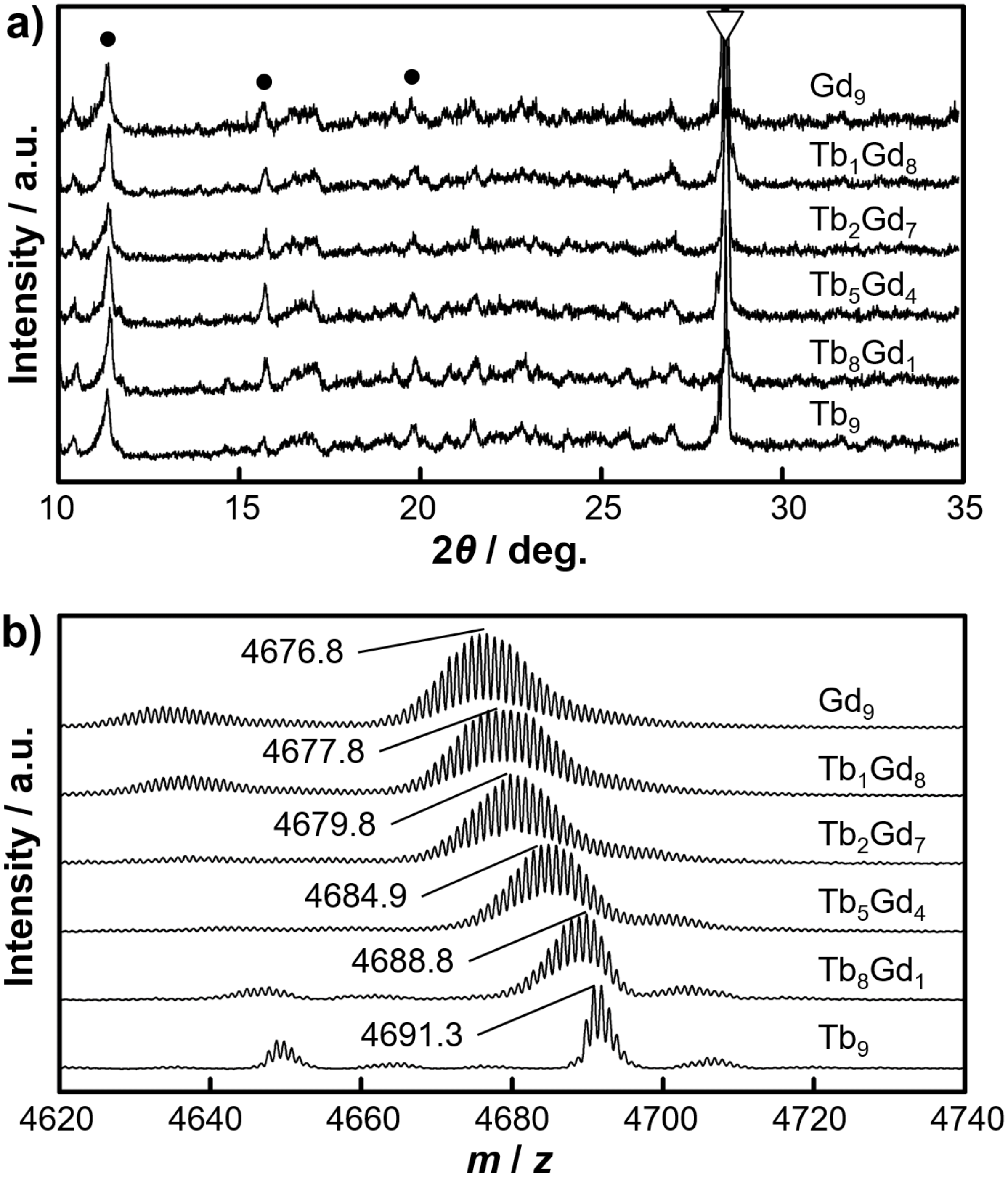

Figure S2. a) Powder XRD pattern of Tb_nGd_{9-n} clusters. Inverse triangle represents peaks of Si power as a standard, and black dots represent the main peaks. b) FAB-MS results of Tb_nGd_{9-n} clusters. Broadening of the peak with increasing number of Gd(III) ions are due to stable isotopes.[11]

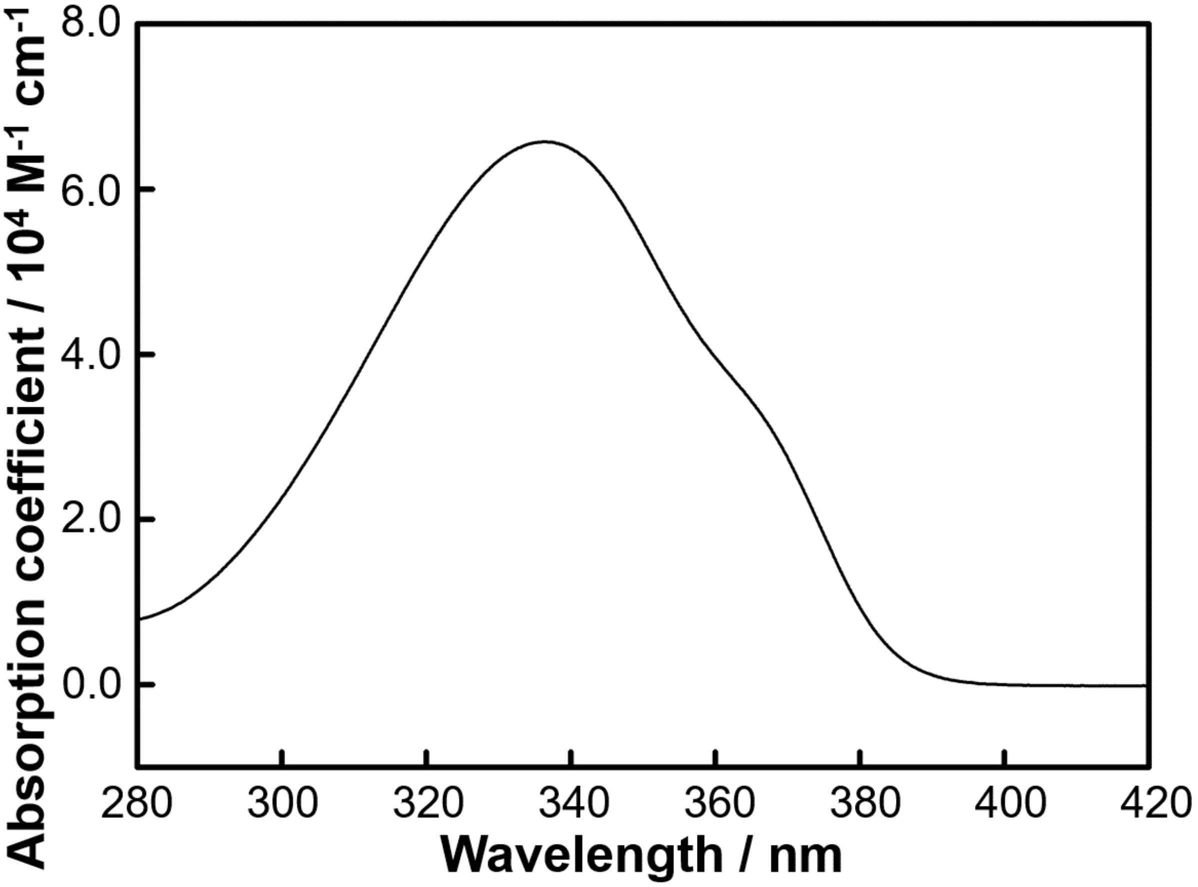

Figure S3. Absorption spectrum of Gd_9 cluster in 1.0×10^{-4} M chloroform solution. 1 mm optical path cell was used for measurement.

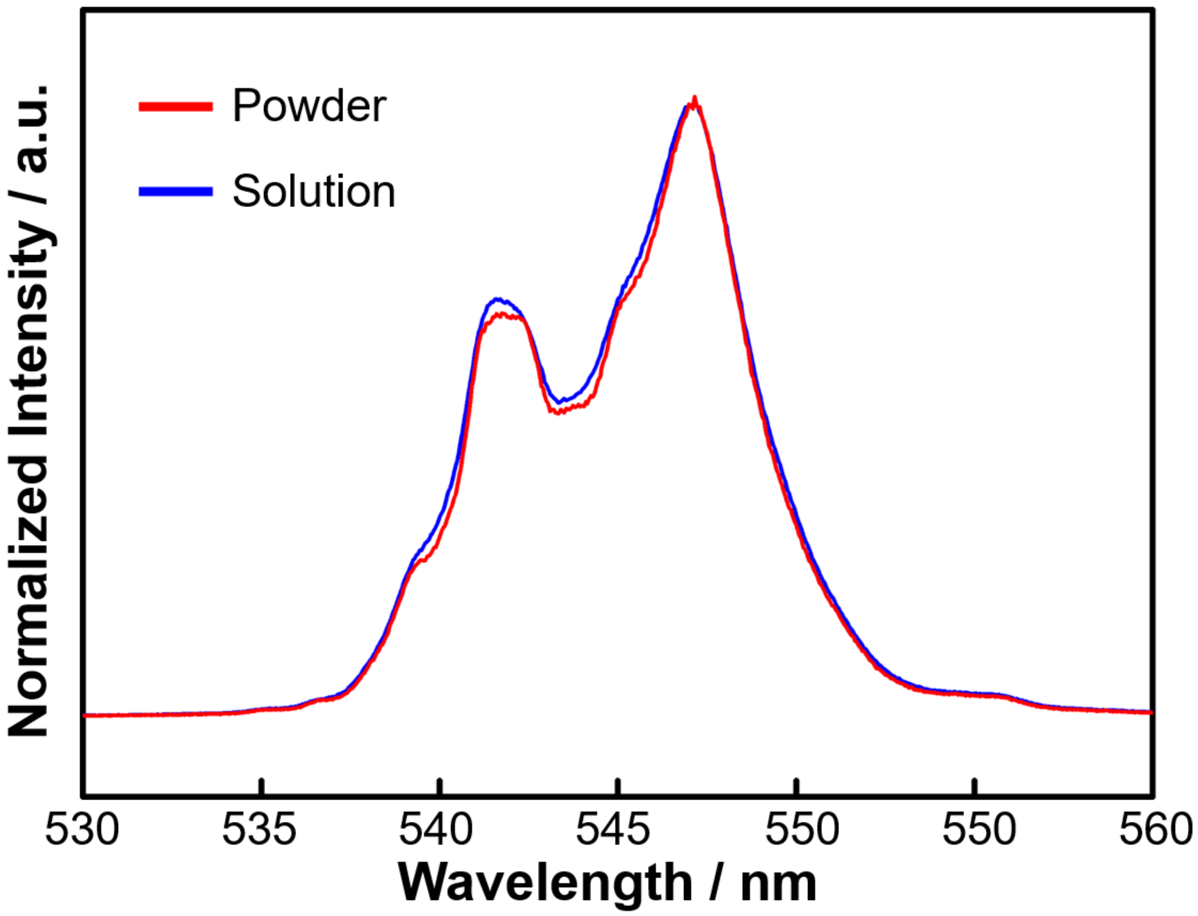

Figure S4. Normalized emission spectra of Tb_9 cluster in powder form and 1.0×10^{-4} M chloroform solution. The spectra were normalized at peak top of $^5D_4 \rightarrow ^7F_5$ transition (548 nm). Excitation wavelength was 380 nm.

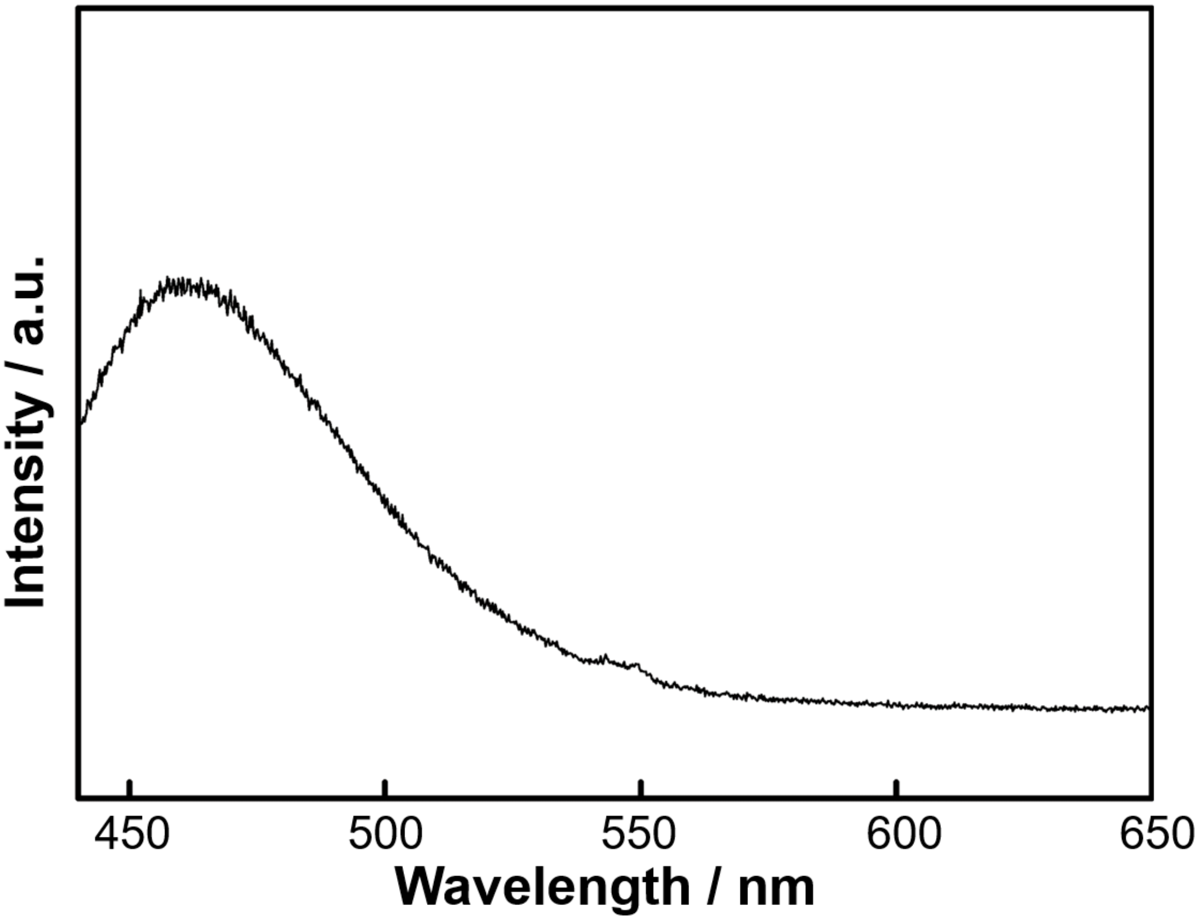

Figure S5. Emission spectrum of Gd_9 cluster in 1.0×10^{-4} M chloroform solution at 210 K. Excitation wavelength was 380 nm.

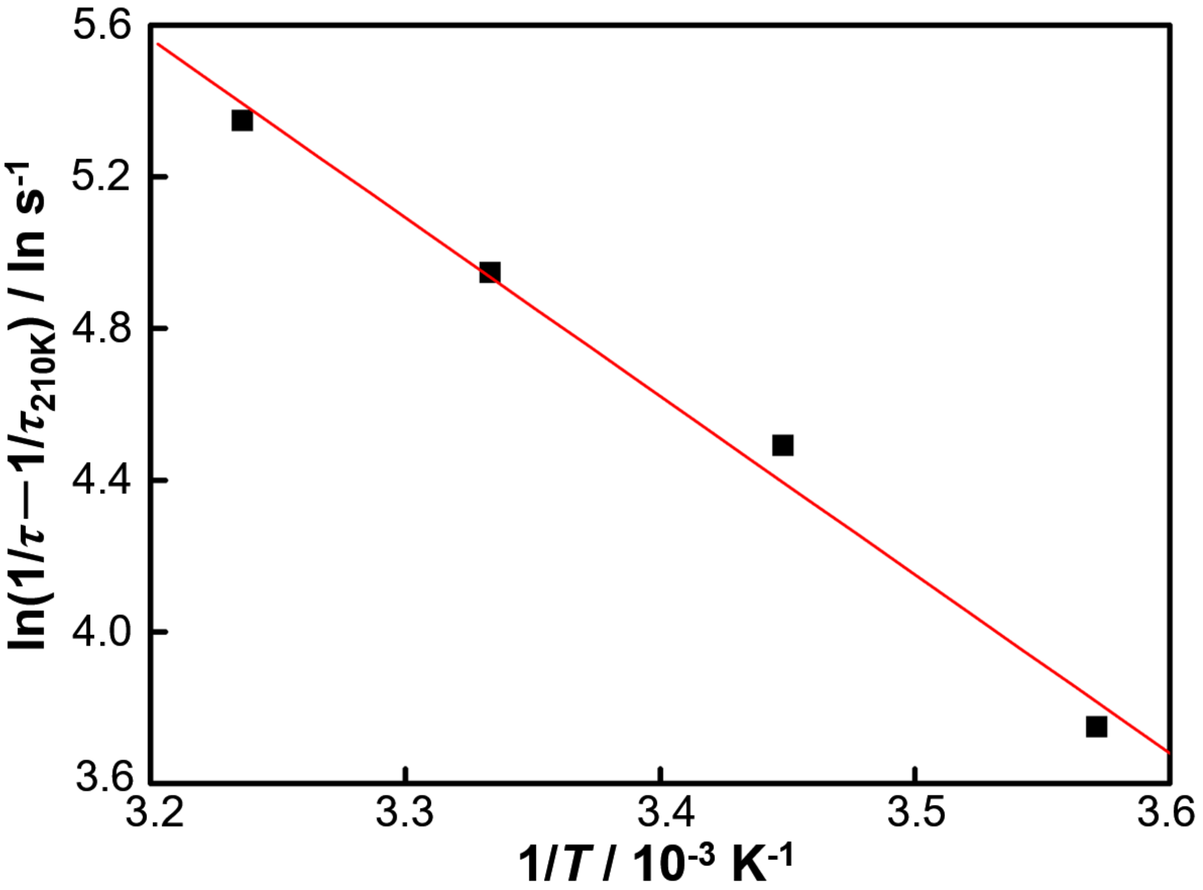

Figure S6. Arrhenius plot of Tb_1Gd_8 cluster from 280－309 K based on Equation (9). Red line represents linearly fitted line. The inclination represents $-\frac{Ea_{\mathrm{BET}}}{R}$, allowing calculation of activation energy of BET.

References

1. Förster, T. 10th Spiers memorial lecture - transfer mechanisms of electronic excitation. *Discuss. Faraday Soc.* 7–17 (1959).
2. Kushida, T. Energy transfer and cooperative optical transitions in rare earth doped inorganic materials. I. Transition probability calculation. *J. Phys. Soc. Japan* **34,** 1318–1326 (1973).
3. Malta, O. L. Mechanisms of non-radiative energy transfer involving lanthanide ions revisited. *J. Non. Cryst. Solids* **354,** 4770–4776 (2008).
4. DiBartolo, B. Energy transfer processes in condensed matter; Plenum Press: New York, (1984).
5. Dexter, D. L. A theory of sensitized luminescence in solids. *J. Chem. Phys.* **21**, 836–850 (1953).
6. Duhamel-Henry, N., Adam, J. L., Jacquier, B. & Linarès, C. Photoluminescence of new fluorophosphate glasses containing a high concentration of terbium (III) ions. *Opt. Mater.* **5,** 197–207 (1996).
7. Yamashita, T. & Ohishi, Y. Concentration and temperature effects on the spectroscopic properties of Tb^{3+} doped borosilicate glasses. *J. Appl. Phys.* **102,** (2007).
8. Casanova, D., Llunell, M., Alemany, P. & Alvarez, S. The rich stereochemistry of eight-vertex polyhedra: a continuous shape measures study. *Chem. Eur. J.* **11**, 1479–1494 (2005).
9. Pinsky, M. & Avnir, D. Continuous symmetry measures. 5. the classical polyhedra. *Inorg. Chem.* **37**, 5575–5582 (1998).
10. Xu, J., Radkov, E., Ziegler, M. & Raymond, K. N. Plutonium(IV) sequestration: structural and thermodynamic evaluation of the extraordinarily stable cerium(IV) hydroxypyridinonate complexes. *Inorg. Chem.* **39**, 4156–4164 (2000).
11. de Laeter, J. R. et al. Atomic weights of the elements: review 2000. *Pure Appl. Chem.* **75**, 683–800 (2003).
